# Withdrawn and wired: Problematic internet use accounts for the link of neurotic withdrawal to sleep disturbances

**DOI:** 10.5935/1984-0063.20180015

**Published:** 2018

**Authors:** Anne Danielle Herlache, Kathryn M. Lang, Zlatan Krizan

**Affiliations:** 1 Internal Revenue Service, Research Division - Washington - D.C. - USA.; 2 Western Carolina University, Psychology - Cullowhee - NC - USA.; 3 Iowa State University, Psychology - AMes - IA - USA.

**Keywords:** Sleep Wake Disorders, Sleep, Internet, Personality

## Abstract

Although neuroticism is the strongest personality predictor of sleep disturbance, it is not clear whether dysphoric (Withdrawal) or angry (Volatility) aspect of neuroticism is more important and whether problematic technology use plays an intervening role. To this end, this study examined distinct contributions of neurotic withdrawal and volatility in predicting self-reported sleep disturbance while testing the mediating role of problematic internet use. **Methods:** One-hundred and fourty-three college students completed an online survey that included measures of neuroticism, sleep quality, and problematic internet use. **Results:** Although both aspects of neuroticism predicted poor sleep, Withdrawal emerged as a stronger and the only unique predictor. Furthermore, problematic internet use explained a portion of Withdrawal’s relationship to worse sleep, especially nighttime and daytime disturbances. **Discussion:** The findings suggest that dysphoric rather than angry features of neuroticism are more important for sleep problems and that the problematic use of modern technology may be an important contributing factor.

## INTRODUCTION

Impaired sleep yields a variety of negative consequences, including impaired vigilance, poor reasoning, and impulse control failures^1^^,^^2^. For these reasons, sleep impairment is detrimental to everyday life, ranging from performance reductions in academic and work settings to increases in mental illness and intimate partner violence^3^^-^^5^. Identifying personality vulnerabilities to sleep impairment and the intervening processes is thus a vital step forward in illuminating ways to lessen the severe burdens that accompanies sleep loss.

Indeed, personality differences in trait anxiety, depression, and neuroticism more generally have been extensively linked to poor sleep quality and future insomnia^6^^-^^8^. Building on these findings, we sought to illuminate this link by investigating which aspect of neuroticism more closely signals poor sleep, namely Withdrawal (anxiety and depression) or Volatility (anger and defensiveness). Moreover, we examined whether problematic internet use contributed to this relationship between neuroticism and sleep problems. Individuals in today’s society use internet more than ever before, with increased mobile technology use being directly implicated in less sleep and sleep of poorer quality^9^. To our knowledge this is the first investigation to link distinct aspects of neuroticism with sleep problems while simultaneously examining the role of internet use.

## NEUROTIC VOLATILITY VS. NEUROTIC WITHDRAWAL

Neuroticism refers to the disposition toward distress and negative emotional states and is characterized by two aspects reflecting anxiety and dysphoria on one hand, and anger with irritability on the other^10^^,^^11^. These core personality aspects of neuroticism are labeled Volatility (“Get angry easily,”) and Withdrawal (“Worry about things”)^10^. These distinct aspects parallel differences between externalizing (e.g., aggressive) and internalizing (e.g., dysphoric) responses to stressors^12^.

Neurotic Volatility reflects behavioral instability and difficulty controlling emotional impulses. It is linked with tendencies toward irritability and anger, while also showing association with externalizing problems^12^. An FMRI study indicated that volatility is associated with sensitivity to threatening stimuli and a general tendency to approach them, even if negative in valence^13^.

In contrast, neurotic Withdrawal involves retreat from threatening events while experiencing negative affect. This negative affect is often evident in self-doubts, a sense of anxiety, and depressive reactions^10^. This leads individuals to remove themselves from whatever situation aroused those responses; for example, neurotic withdrawal was linked to a pattern of amygdala activation indicating a predisposition toward avoidance of threatening stimuli, rather than approach^13^. In this vein, it is thought to be more closely associated with the behavioral *inhibition* system (as opposed to the behavioral approach system)^10^. In short, neuroticism reflects a general sensitivity to threatening stimuli, although the expression of that sensitivity is a combination of distinct tendencies toward volatility and withdrawal.

## IS WITHDRAWAL OR VOLATILITY MORE IMPORTANT FOR SLEEP?

For individuals prone to emotional instability, anxiety, and worry, such tendencies may be particularly troublesome when distractions aren’t readily available, such as when trying to sleep. In this vein, neuroticism is predictive of sleep onset problems both in adolescence (*r*=.34) and adulthood (*r*=.25)^14^. A study that used actigraphy to track sleep also confirmed that adolescents who felt the hallmarks of neuroticism during the day (e.g., anxiety, nervousness, irritability) slept objectively fewer hours and had longer awakenings that night^15^. Moreover, an analysis of more than 22,000 individuals that neuroticism was both concurrently and prospectively associated with worsening sleep quality^16^. Although this evidence clearly implicates neuroticism in sleep problems, there is little data on which aspect of neuroticism (volatility or withdrawal) is more important.

On one hand, Volatility involves externalizing problems (e.g., short-lived angry outbursts) and should be linked to poorer sleep quality through a connection with chronic anger and conflict^5^. On the other hand, Withdrawal is associated with loneliness, depression, and anxiety, all of which have been strongly and consistently tied to sleep problems and development of insomnia^8^^,^^10^^,^^17^. In the current study we tested the unique and relative importance of these aspects of neuroticism for sleep disturbances. Moreover, we investigated the intervening role of an increasingly important factor for sleep, namely internet use.

## THE ROLE OF PROBLEMATIC INTERNET USE

Problematic internet use (uncontrollable use that leads to social-occupational problems) has been extensively linked to psychiatric and behavioral problems in adolescents and young adults, with more neurotic individuals being especially prone to compulsive or extreme internet use^18^^,^^19^. Similarly, problematic internet use may delay sleep onset and it predicts poor sleep quality^20^.

However, it is not clear whether pathological internet use is more relevant to Withdrawal or Volatility when it comes to individuals’ sleep. On one hand, compulsive and uncontrolled use of the internet can be driven both by general problems with impulse control and reward-seeking behavior (implicating volatility), as well as desires to escape personal distress or loneliness (implicating withdrawal^21^). Given the strong ties between problematic internet use, anxiety, depression, and sleep disturbance, we anticipated that problematic internet use would mainly contribute to the link between Withdrawal and sleep problems, contributing less to any link between Volatility and poor sleep. In line with this premise, one study found that people high in neuroticism were motivated to pursue online activities in an attempt to escape loneliness^22^. Importantly, loneliness is also linked to worse sleep quality^23^. Thus, pursuit of online activities can be a manifestation of withdrawal and coping with negative affect, in that it allows a person to manage their exposure to potentially threatening stimuli (e.g., avoiding unwanted conversations).

## THE CURRENT STUDY

As internet usage becomes ubiquitous, it is necessary to understand its role in tying personality differences to key aspects of health such as nightly sleep disturbances and daily fatigue. To this end, the current study examined the links among neuroticism, problematic internet use, and sleep in a sample of college students. Given the stability of personality traits and their role in affecting sleep, we hypothesized a model rooted in neuroticism predicting problematic internet usage and sleep quality. To our knowledge, this is the first direct examination of the role that problematic internet use plays in tying different aspects of neuroticism to sleep quality.

First, although we anticipated withdrawal and volatility both to predict sleep quality, we expected withdrawal to show a stronger unique relation given extensive ties between anxiety, depression, and sleep problems. Furthermore, we expected that problematic internet use would predict sleep disturbances and that it would help account for the link between Withdrawal and worse sleep, but not between Volatility and worse sleep. While cross-sectional in nature, this study sheds light on important differences in aspects of neuroticism as they relate to problematic internet use and sleep health.

## METHODS

### Participants and Procedure

A power analysis using G*Power indicated that 144 observations would be sufficient to capture a moderate correlation of .27 at .05 level of significance, approximating correlations between neuroticism and sleep quality reported in prior research^14^. To this end, 143 undergraduate students from a large U.S. university participated in a large, online study for course credit (age 18-36, *M*=19.85, *SD*=2.45; 38.3% female; 76.8% White, 12.7% Asian/Pacific Islander). They completed a series of surveys relevant to sleep and personality. No other measures of neuroticism, problematic internet use, or sleep quality were administered except those described in this report. The study was approved by the local Research Ethics Committee and all participants provided informed consent.

The measures used in the current study were the *Big Five Aspect Scale of Volatility (*10 items, α=.859, sample item: “Get easily agitated”) and *Withdrawal (*10 items, α=.721, “Become overwhelmed by events”^10^ ^10^), the *Problematic Internet Use Questionnaire* (37 items, α=.965; “Have you ever tried to escape your problems by going online?”)^24^, and *the Pittsburgh* *Sleep Quality Index,* (PSQI)^25^. Sleep quality on this measure reflects three distinct factors, namely *Sleep Efficiency* (time asleep relative to time in bed), *Perceived Sleep Quality* (reported integrity of sleep), and *Daily Disturbances* (fatigue and sleep interruptions)^26^. No information regarding psychiatric disorders or medication use was collected.

### Statistical Analyses

We first examined bivariate correlations between the variables of interest to appraise the relations between aspects of neuroticism, problematic internet use, and sleep (Table 1). We then tested a path model with bootstrapping in Mplus (v.7) with neuroticism aspects predicted features of sleep quality via problematic indirect use. Besides total effects linking neuroticism aspects and sleep problems, of key interests were the *indirect* effects between distinct aspects of neuroticism and sleep quality through problematic internet use.

**Table 1:** Correlations among measures of neuroticism, sleep, and problematic internet use.

	1	2	3	4	5	6
1. Neuroticism						
2. Withdrawal	.82**					
3. Volatility	.89**	.46**				
4. Problematic Internet Use	.34**	.36**	.22**			
5. Daily Sleep Disturbance	.32**	.38**	.19*	.45**		
6. Sleep Efficiency	.07	.08	.05	.16*	.17*	
7. Perceived Sleep Quality	.31**	.39**	.17*	.16	.37**	.25**

Note:

** *p*<.01,

* *p*<.05.

## RESULTS

As anticipated, neuroticism generally predicted worse sleep quality and more problematic internet use (Table 1). Basic correlations also revealed that Withdrawal was more strongly related to both daily disturbances and sleep quality than was Volatility, in accord with our first hypothesis (neither aspect of neuroticism was significantly correlated with self-reported sleep efficiency). In short, these correlations support the hypothesis Withdrawal is more important for problems with sleep and internet use than is Volatility, although both matter.

**Figure 1:**
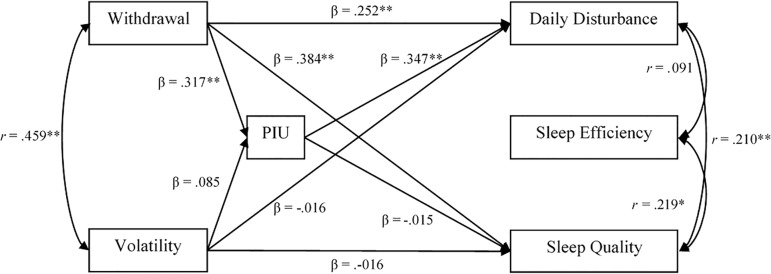
Neuroticism Aspects as Predictors of Sleep Disturbances via Problematic Internet Use ** *p* <.01, * *p* <.05. PIU = Problematic Internet Use. Indirect path from Withdrawal to Daily Disturbance (b = .11, *p* = .010). All other indirect paths failed to reach conventional standards for significance. Enviado por: Zlatan Krizan Krizan.

To test our second hypothesis, we further tested the intervening role of problematic internet use in the relationship between neuroticism and sleep in a model regressing the three sleep quality factors on problematic internet use and the two aspects of neuroticism. We focused the analysis on sleep quality and daily disturbances as correlations indicated that sleep efficiency had little association with neuroticism or problematic internet use (Table 1). This model yielded good fit to the data (χ^2^(3)=4.470, *p*=.215, RMSEA=.059, CFI=.984, Figure 1).

As expected, the Withdrawal subscale of neuroticism accounted for more variability in problematic internet use than did volatility (Withdrawal: *β=.3*2, *p*<.001, 95% CI=.16, .47; Volatility: *β=.*09, *p*=.284, CI=-.08, .24). Overall inspection of the links between the aspects of neuroticism and sleep again reveals that Withdrawal played the stronger role *(Daily Disturbance*: Withdrawal, *β=.*25, *p*=.001, 95% CI=.11, .40; Volatility, *β =* -.02, *p* = .84, CI = -.17, .13; *Sleep Quality*: Withdrawal, *β=.*38, *p*=.001, 95% CI=.21, .57; Volatility, *β=*-.02, *p*=.881, CI=-.24, .20). Likewise, problematic internet use predicted sleep problems, specifically daily disturbances (β=.35, *p*<.001, 95% CI=.16, .52; *Sleep Quality*, β=-.02, *p*=.864, 95% CI=-.19, .16). Importantly, such use partially accounted for the relationship between Withdrawal and Daily Disturbances (β Indirect=.11, *p*=.010, 95% CI=.04, .22), whereas there was no such indirect link for Volatility (β Indirect=.03, *p*=.29, 95% CI=-.02, .09). Taken together, these data support our hypotheses that a) Withdrawal is more indicative of sleep problems than Volatility and that b) problematic internet use plays an intervening role in the relationship between neurotic withdrawal and poor sleep, especially nighttime and daytime disturbances in sleep.

## DISCUSSION

The present study investigated a) whether aspects of neuroticism differently related to sleep quality, b) whether Problematic Internet Use was associated with sleep problems, and c) whether problematic internet use contributed to the relation between distinct aspects of neuroticism and sleep. Although the tested model is not causal, our results suggest that personality tendencies toward withdrawal fuel problematic internet use that contributes to concomitant sleep problems and their impact on daily functioning.

Although Volatility was correlated with both daytime and nigh-time sleep problems, these relationships disappeared when Withdrawal and problematic internet use were taken into account. In contrast, Withdrawal emerged as strongly and uniquely linked with all of these variables, regardless of Volatility. Also, it appears that problematic internet use only contributed to Withdrawal’s relationship with reported disturbances, but not with perceived sleep quality. This indicates that for individuals higher on Withdrawal, problematic internet use is more indicative of fragmented and dysregulated sleep, rather than of direct perceptions of poor sleep^26^. Although more compulsive internet use may delay and displace sleep, these results suggest such use may be especially important for the integrity of sleep and daytime functioning, and especially among those prone to neurotic withdrawal. Future research should thus directly focus on identifying aspects of sleep that are most harmed by problematic use, especially in adolescence that involves frequent use of technology^20^.

There are also limitations to this study. The data were cross-sectional in nature and the analyses are not definitive tests of any causal relations. Although it is unlikely that current sleep and internet use affected personality traits, this study cannot definitively determine whether internet use affected individuals’ sleep or vice versa. Indeed, prospective evidence indicates that developing internet addiction leads to more hostility and depression over a year, suggesting problematic internet use can increase neuroticism over the long-term^27^. A longitudinal examination of neuroticism in the context of internet use and sleep disturbances would allow study of patterns in day-to-day functioning, including reciprocal relationships. Nevertheless, the current study is novel in its attempt to identify factors that help explain the role of neuroticism in sleep impairment.

To our knowledge this is the first study to investigate the role of problematic internet use in that relationship. To people for whom in-person interactions feel threatening, an online escape may seem preferable-particularly when rumination on daytime interactions is likely (i.e., while attempting to sleep). Withdrawal appears to prompt people to seek the comforts they think an online experience can provide, potentially when they might otherwise be seeking sleep. Furthermore, troubles stemming from withdrawal and problematic internet use can persist into the day, evident in fatigue and potentially lower performance in daytime activities. Understanding such vulnerabilities provides a foundation for developing strategies to ameliorate barriers to quality sleep. For example, addressing the underlying anxieties experienced by people prone to Withdrawal could improve their sleepiness, possibly by lowering the amount of online distraction they seek.

## References

[1] Thomas M, Sing H, Belenky G, Holcomb H, Mayberg H, Dannals R (2000). Neural basis of alertness and cognitive performance impairments
during sleepiness. I. Effects of 24 h of sleep deprivation on waking human
regional brain activity. J Sleep Res.

[2] Lim J, Dinges DF (2010). A meta-analysis of the impact of short-term sleep deprivation on
cognitive variables. Psychol Bull.

[3] Taylor DJ, Vatthauer KE, Bramoweth AD, Ruggero C, Roane B (2013). The role of sleep in predicting college academic performance: is
it a unique predictor?. Behav Sleep Med.

[4] Hoshino K, Pasqualini JC, D’Oliveira EP, Silva CP, Modesto AE, Silveira RSM (2009). Is sleep deprivation involved in domestic
violence?. Sleep Sci.

[5] Krizan Z, Herlache AD (2016). Sleep disruption and aggression: Implications for violence and
its prevention. Psychol Violence.

[6] Calkins AW, Hearon BA, Capozzoli MC, Otto MW (2013). Psychosocial predictors of sleep dysfunction: the role of anxiety
sensitivity, dysfunctional beliefs, and neuroticism. Behav Sleep Med.

[7] Duggan KA, Friedman HS, McDevitt EA, Mednick SC (2014). Personality and healthy sleep: The importance of
conscientiousness and neuroticism. PloS One.

[8] Singareddy R, Vgontzas AN, Fernandez-Mendoza J, Liao D, Calhoun S, Shaffer ML (2012). Risk factors for incident chronic insomnia: a general population
prospective study. Sleep Med.

[9] Twenge J, Krizan Z, Hisler G (2017). Decreases in self-reported sleep duration among U.S. adolescents
2009-2015 and association with new media screen time. Sleep Med.

[10] DeYoung CG, Quilty LC, Peterson JB (2007). Between facets and domains: 10 aspects of the Big
Five. J Pers Soc Psychol.

[11] Saucier G, Goldberg LR, Institute OR (2001). Lexical studies of indigenous personality factors: premises,
products, and prospects. J Pers.

[12] Kessler RC, Petukhova M, Zaslavsky AM (2011). The role of latent internalizing and externalizing
predispositions in accounting for the development of comorbidity among
common mental disorders. Curr Opin Psychiatry.

[13] Cunningham WA, Arbuckle NL, Jahn A, Mowrer SM, Abduljalil AM (2010). Aspects of neuroticism and the amygdala: chronic tuning from
motivational styles. Neuropsychologia.

[14] Danielsson NS, Jansson-Fröjmark M, Linton SJ, Jutengren G, Stattin H (2010). Neuroticism and sleep-onset: What is the long-term
connection?. Pers Individ Dif.

[15] Tavernier R, Choo SB, Grant K, Adam EK (2016). Daily affective experiences predict objective sleep outcomes
among adolescents. J Sleep Res.

[16] Stephan Y, Sutin AR, Bayard S, Križan Z, Terracciano A (2018). Personality and subjective sleep quality: Evidence from four
prospective studies. Health Psychol.

[17] Lovato N, Gradisar M (2014). A meta-analysis and model of the relationship between sleep and
depression in adolescents: recommendations for future research and clinical
practice. Sleep Med Rev.

[18] Kayiş AR, Satici SA, Yilmaz FM, Şimşek D, Ceyhan E, Bakioğlu F (2016). Big-five personality trait and internet addiction: A
meta-analytic review. Comput Human Behav.

[19] Shapira NA, Goldsmith TD, Keck Jr PE, Khosla UM, McElroy SL (2000). Psychiatric features of individuals with problematic internet
use. J Affect Disord.

[20] Bruni O, Sette S, Fontanesi L, Baiocco R, Laghi F, Baumgartner E (2015). Technology Use and Sleep Quality in Preadolescence and
Adolescence. J Clin Sleep Med.

[21] De Leo JA, Wulfert E (2013). Problematic Internet use and other risky behaviors in college
students: an application of problem-behavior theory. Psychol Addict Behav.

[22] Amiel T, Sargent SL (2004). Individual differences in internet usage motives. Comput Human Behav.

[23] McHugh J, Lawlor B (2011). Living alone does not account for the association between
loneliness and sleep in older adults: Response to Hawkley, Preacher, and
Cacioppo, 2010. Health Psychol.

[24] Thatcher A, Goolam S (2005). Development and psychometric properties of the Problematic
Internet use Questionnaire. S Afr J Psychol.

[25] Buysse DJ, Reynolds 3rd CF, Monk TH, Berman SR, Kupfer DJ (1989). The Pittsburgh Sleep Quality Index: A new instrument for
psychiatric practice and research. Psychiatry Res.

[26] Cole JC, Motivala SJ, Buysse DJ, Oxman MN, Levin MJ, Irwin MR (2006). Validation of a 3-factor scoring model for the Pittsburgh sleep
quality index in older adults. Sleep.

[27] Ko CH, Liu TL, Wang PW, Chen CS, Yen CF, Yen JY (2014). The exacerbation of depression, hostility, and social anxiety in
the course of Internet addiction among adolescents: a prospective
study. Compr Psychiatry.

